# Supplementary material for: Indirect benefits of seasonal malaria chemoprevention for non-malarial pediatric infections and routine antibiotic use in real-world programmatic settings: a pre-post study using positive and negative controls
Source: BMC Infect Dis. 2026 Jan 12;26:296. doi: 10.1186/s12879-026-12528-y (PMC12888588; doi:10.1186/s12879-026-12528-y)
Supplement: Supplementary file 1 — Supplementary Material 1 [file 12879_2026_12528_MOESM1_ESM.docx]

**Supplementary Figure 1-** Diagnoses Rates and Treatment Prescription Rates for Control Outcomes in 2020 and 2021 in the Context of SMC Administration


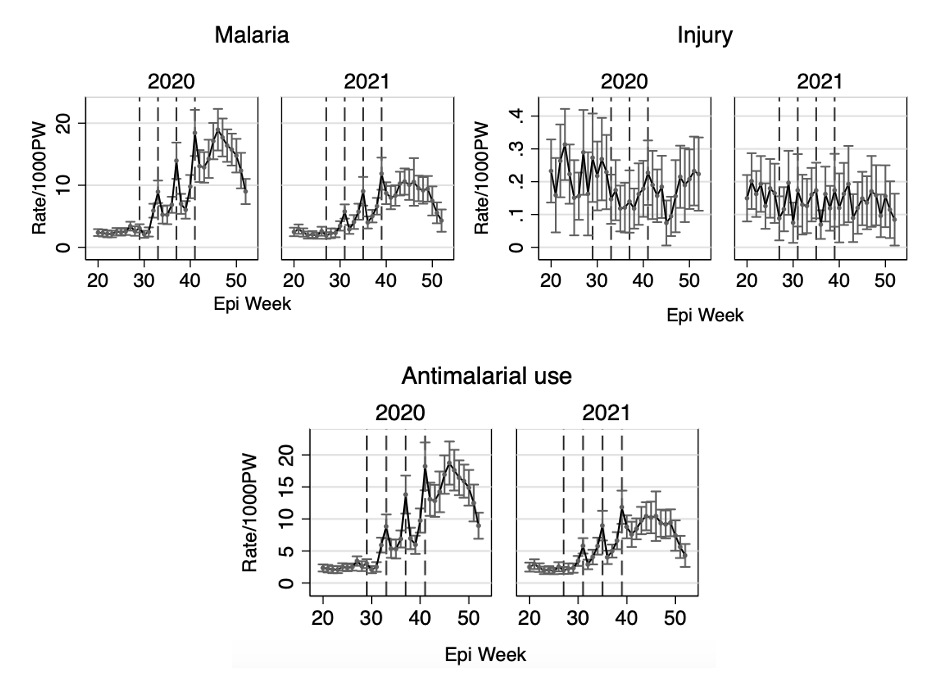


| **Supplementary Table 1-** Changes in Diagnoses and Treatment rates following SMC by Month of SMC Administration and Overall (IRR, 95% CI) | | | | | | |
| --- | --- | --- | --- | --- | --- | --- |
| **Outcome** | **Overall** | **July** | **August** | **September** | **October** | **P value for global test of interaction** |
| Pneumonia | 0.86 (0.79 to 0.93) | 0.66 (0.57 to 0.74) | 0.92 (0.80 to 1.05) | 1.0 (0.83 to 1.18) | 0.81 (0.69 to 0.92) | 0.000 |
| Diarrhea | 0.83 (0.74 to 0.93) | 1.02 (0.82 to 1.22) | 0.74 (0.61 to 0.88) | 0.85 (0.70 to 1.0) | 0.82 (0.67 to 0.97) | 0.047 |
| Acute malnutrition | 0.71 (0.51 to 0.97) | 0.49 (0.03 to 0.94) | 0.69 (0.23 to 1.14) | 0.81 (0.29 to 1.34) | 0.80 (0.47 to 1.13) | 0.654 |
|  |  |  |  |  |  |  |
| Malaria  (positive control) | 0.62 (0.56 to 0.69) | 0.90 (0.73 to 1.07) | 0.59 (0.52 to 0.67) | 0.54 (0.47 to 0.61) | 0.67 (0.58 to 0.75) | 0.000 |
| Injury  (negative control) | 0.89 (0.67 to 1.19) | 0.92 (0.46 to 1.37) | 1.03 (0.57 to 1.48) | 0.80 (0.44 to 1.16) | 0.82 (0.43 to 1.20) | 0.861 |
|  |  |  |  |  |  |  |
| Antibiotic prescription | 0.88 (0.83 to 0.94) | 0.78 (0.70 to 0.86) | 0.92 (0.84 to 1.0) | 1.0 (0.86 to 1.13) | 0.81 (0.72 to 0.91) | 0.002 |
| Antimalarial prescription  (positive control) | 0.63 (0.56 to 0.69) | 0.90 (0.72 to 1.07) | 0.60 (0.53 to 0.68) | 0.54 (0.47 to 0.61) | 0.67 (0.58 to 0.76) | 0.000 |

**Supplementary Figure 2**- Difference in Incidence Rates of Diagnoses and Treatments During SMC Administration vs.

Post-Administration Weeks, by Month of Administration for Control Outcomes


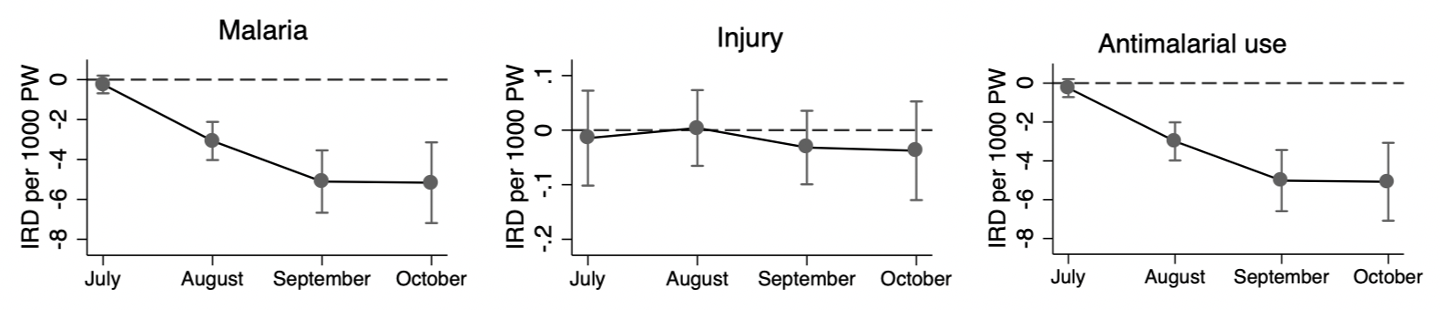


Note: P-values from the global test of interaction between SMC and month of administration were 0.000 and 0.861 for malaria

and injury, respectively, and 0.001 for antimalarial prescription rates

**Supplementary Table 2-** Incidence Rates, Rate Ratios, and Rate Differences of Outcomes During SMC Administration Weeks and the First, Second-, and Third-Weeks Post-Administration

| **Weeks post SMC administration** | **Incidence rate per 1,000 person-week (95%CI)** | **Incidence rate ratio (95%CI)** | **Incidence Rate Difference per 1,000 person-week (95%CI)** |
| --- | --- | --- | --- |
| Malaria (positive control) |  |  |  |
| Admin week | 9.7 (8 to 11.4) | Ref | Ref |
| 1 | 6.0 (4.9 to 7.1) | 0.61 (0.55 to 0.68) | -3.8 (-4.8 to -2.8) |
| 2 | 5.8 (4.9 to 6.8) | 0.6 (0.53 to 0.68) | -3.9 (-5.1 to -2.7) |
| 3 | 6.4 (5.4 to 7.3) | 0.65 (0.59 to 0.73) | -3.4 (-4.5 to -2.3) |
| Injury (negative control) |  |  |  |
| Admin week | 0.17 (0.12 to 0.23) | Ref | Ref |
| 1 | 0.14 (0.11 to 0.17) | 0.81 (0.58 to 1.13) | -0.03 (-0.09 to 0.02) |
| 2 | 0.17 (0.13 to 0.21) | 0.98 (0.73 to 1.31) | 0.0 (-0.06 to 0.05) |
| 3 | 0.15 (0.11 to 0.2) | 0.89 (0.63 to 1.26) | -0.02 (-0.08 to 0.04) |
| Pneumonia |  |  |  |
| Admin week | 4.7 (3.8 to 5.7) | Ref | Ref |
| 1 | 4.3 (3.5 to 5) | 0.9 (0.83 to 0.97) | -0.5 (-0.9 to -0.1) |
| 2 | 4.1 (3.2 to 4.9) | 0.86 (0.78 to 0.95) | -0.7 (-1.1 to -0.2) |
| 3 | 3.9 (3.1 to 4.6) | 0.82 (0.74 to 0.90) | -0.9 (-1.3 to -0.4) |
| Diarrhea |  |  |  |
| Admin week | 1.4 (1 to 1.7) | Ref | Ref |
| 1 | 1.2 (0.9 to 1.5) | 0.87 (0.77 to 0.98) | -0.2 (-0.3 to 0) |
| 2 | 1.0 (0.8 to 1.3) | 0.77 (0.66 to 0.90) | -0.3 (-0.5 to -0.1) |
| 3 | 1.2 (0.9 to 1.5) | 0.86 (0.76 to 0.97) | -0.2 (-0.3 to 0) |
| Acute Malnutrition |  |  |  |
| Admin week | 0.17 (0.09 to 0.24) | Ref | Ref |
| 1 | 0.12 (0.05 to 0.2) | 0.74 (0.49 to 1.11) | -0.04 (-0.1 to 0.01) |
| 2 | 0.10 (0.04 to 0.17) | 0.63 (0.42 to 0.94) | -0.06 (-0.11 to -0.01) |
| 3 | 0.13 (0.06 to 0.19) | 0.76 (0.54 to 1.06) | -0.04 (-0.09 to 0.01) |
| Antimalarial prescription (positive control) |  |  |  |
| Admin week | 9.7 (7.9 to 11.4) | Ref | Ref |
| 1 | 6 (4.8 to 7.1) | 0.62 (0.56 to 0.69) | -3.7 (-4.7 to -2.7) |
| 2 | 5.8 (4.8 to 6.8) | 0.60 (0.53 to 0.68) | -3.9 (-5.1 to -2.7) |
| 3 | 6.4 (5.4 to 7.4) | 0.66 (0.59 to 0.74) | -3.3 (-4.4 to -2.2) |
| Antibiotic prescription |  |  |  |
| Admin week | 7.9 (6.6 to 9.2) | Ref | Ref |
| 1 | 7.1 (5.8 to 8.3) | 0.90 (0.84 to 0.96) | -0.82 (-1.33 to -0.31) |
| 2 | 6.9 (5.7 to 8) | 0.87 (0.81 to 0.93) | -1.02 (-1.57 to -0.46) |
| 3 | 6.9 (5.7 to 8.1) | 0.87 (0.8 to 0.95) | -0.99 (-1.61 to -0.36) |
